# Genetic ablation or pharmacological blockade of dipeptidyl peptidase IV does not impact T cell-dependent immune responses

**DOI:** 10.1186/1471-2172-10-19

**Published:** 2009-04-09

**Authors:** Kalpit A Vora, Gene Porter, Roche Peng, Yan Cui, Kellyann Pryor, George Eiermann, Dennis M Zaller

**Affiliations:** 1 Department of Immunology, Merck Research Laboratories, PO Box 2000, Rahway, New Jersey 07065, USA; 2 Department of Metabolic Disorder, Merck Research Laboratories, PO Box 2000, Rahway, New Jersey 07065, USA; 3 Department of Pharmacology, Merck Research Laboratories, PO Box 2000, Rahway, New Jersey 07065, USA; 4 Schering-Plough Research Institute, 2015 Galloping Hill Road, K15-2-D224C-2700, Kenilworth, New Jersey 07016, USA

## Abstract

**Background:**

Current literature suggests that dipeptidyl peptidase IV (DPP-IV; CD26) plays an essential role in T-dependent immune responses, a role that could have important clinical consequences. To rigorously define the role of DPP-IV in the immune system, we evaluated genetic and pharmacological inhibition of the enzyme on T-dependent immune responses *in vivo*.

**Results:**

The DPP-IV null animals mounted robust primary and secondary antibody responses to the T dependent antigens, 4-hydroxy-3-nitrophenylacetyl-ovalbumin (NP-Ova) and 4-hydroxy-3-nitrophenylacetyl-chicken gamma globulin (NP-CGG), which were comparable to wild type mice. Serum levels of antigen specific IgM, IgG1, IgG2a, IgG2b and IgG3 were similar between the two groups of animals. DPP-IV null animals mounted an efficient germinal center reaction by day 10 after antigen stimulation that was comparable to wild type mice. Moreover, the antibodies produced by DPP-IV null animals after repeated antigenic challenge were affinity matured. Similar observations were made using wild type animals treated with a highly selective DPP-IV inhibitor during the entire course of the experiments. T cell recall responses to ovalbumin and MOG peptide, evaluated by measuring proliferation and IL-2 release from cells isolated from draining lymph nodes, were equivalent in DPP-IV null and wild type animals. Furthermore, mice treated with DPP-IV inhibitor had intact T-cell recall responses to MOG peptide. In addition, female DPP-IV null and wild type mice treated with DPP-IV inhibitor exhibited normal and robust *in vivo* cytotoxic T cell responses after challenge with cells expressing the male H-Y minor histocompatibility antigen.

**Conclusion:**

These data indicate Selective inhibition of DPP-IV does not impair T dependent immune responses to antigenic challenge.

## Background

DPP-IV (CD26) is a cell surface 110 kDa glycoprotein expressed on epithelial cells and leukocyte subsets possessing dipeptidyl peptidase activity. [1]. The DPP-IV enzyme is known to cleave the N-terminal dipeptide from the incretin hormones glucagon-like peptide-1 (GLP-1) and glucose-dependent insulinotropic polypeptide (GIP). This cleavage inactivates the hormones thereby neutralizing their prandial insulinotropic effect [2,3]. Targeting the dipeptidyl peptidase activity with low molecular weight enzyme inhibitors restores incretin activity and has led to the successful development of a DPP-IV inhibitor, sitagliptin, as an effective therapy for Type 2 diabetes [3]. A concern regarding the potential for DPP-IV inhibitors to affect immune function and increase infection rates has been raised [4,5], although a recently published analysis of safety using pooled source data showed no significant difference in the incidence of overall or specific types of infection [6].

The role of DPP-IV enzymatic activity in immune function has not been extensively studied, however there are a few reports suggesting that DPP-IV can modulate immune responses [7,8]. Cell culture studies have implicated DPP-IV as a co-receptor in T cell activation [1]. In addition, DPP-IV may affect leukocyte trafficking via cleavage of certain chemokines such as SDF-1 [9]. DPP-IV null animals were shown have reduced humoral immune responses to pokeweed mitogen [10]. In an Ova asthma model, rats expressing a truncated inactive form of DPP-IV due to a genetic polymorphism were shown to have reduced T cell recruitment to the lungs and decreased Ova-specific IgE titers [11]. However, studies with DPP-IV deficient animals do not directly address the role of the dipeptidyl peptidase activity as this cell surface protein may possess other non-enzymatic functions [12-14]. In addition, some reports that attributed immunomodulatory effects to DPP-IV enzymatic activity may have been confounded by use of non-selective inhibitors. Indeed, we have previously shown that blockade of T cell activation *in vitro *correlates with inhibitor activity directed against DPP8/9 but not against DPP-IV [15]. Moreover, inhibitors that were previously reported to modulate T cell responses were found to be potent inhibitors of DPP8/9 activity [16-21].

To extend these observations to an *in vivo* setting in order to better characterize any potential role of DPP-IV in immune function, we investigated the T cell-dependent responses in mice using genetic ablation or pharmacological blockade of DPP-IV. T cell-dependent antibody responses provides a useful model for addressing immune competence as it is dependent on many factors such as antigen processing and presentation, CD4 T cell help, germinal center reactions, B cell activation and differentiation, affinity maturation, and memory cell formation. We report here that genetic ablation or specific inhibition of DPP-IV did not impair T cell-dependent antibody responses. In addition, we find that genetic ablation or specific inhibition of DPP-IV did not compromise cytotoxic T cell function *in vivo*.

## Methods

### Mice

Female 8 week old C57Bl/6J and DPP-IV^-/- ^[22] mice were obtained from Taconic Laboratory, (Taconic Laboratories, Tarrytown, NY, USA). The DPP-IV^-/- ^mice were originally obtained from Dr. D Marguet and backcrossed on C57BL/6 to homogeneity [23]. SNP testing carried out revealed 98.4% B6J background. The knock out animals were generated by mating male and female homozygous null animals. The control animals were age-matched and obtained from the same facility as the null animals. Animal were housed in a specific pathogen-free rodent facility. All animal protocols were approved by the Merck Institutional Animal Care and Use Committee.

### Antibodies and reagents

To quantify mouse immunoglobulins by ELISA, the following secondary antibodies were used as per manufactures instructions: Rat anti-mouse lambda-Biotin, anti-mouse kappa-Biotin, Rat anti-mouse IgG1-Biotin (BD Biosciences, San Jose, CA, USA), Goat anti-mouse IgG2a-Biotin, Rat anti-mouse IgG2b-Biotin, Goat anti-mouse IgG3-Biotin, and Rat anti-mouse IgM-Biotin (Southern Biotechnology Associate Inc., Birmingham, AL, USA). (4-hydroxy-3-nitrophenyl) acetyl-chicken γ-globulin (NP-CGG, NP-BSA and Ovalbumin) were obtained from Biosearch Technologies, Novato, CA, USA.

MOG p35–55 was obtained from Sigma-Aldrich, St. Louis, MO, USA. Heat-killed *Mycobacterium tuberculosis *was obtained from BD Diagnostics, Franklin Lakes, New Jersey, USA. Pertussis toxin was obtained from List Biological Laboratories, Campbell, CA, USA. The highly selective DPP-IV inhibitor, des-fluro sitagliptin, was synthesized as previously described [24]. To deliver an effective dose of ~400 mg/kg daily, mice were fed a diet consisting of 6.7 g of this compound per 1 kg Tekland chow (Research diets, New Jersey, USA). The enzyme activity of DPP-IV in the blood was assayed as described earlier [15].

### T cell-dependent antibody responses

Mice were immunized i.p. with 100 μg of NP-CGG in alum for the primary immunization, and 100 μg of NP-CGG in PBS i.p. for the secondary immunization [25]. Mice were bled via the retro orbital sinus at indicated times and the levels of anti-NP and CGG antibodies and their isotypes were determined by ELISA as described previously [26,27]. Briefly, mouse serum antibodies were immobilized onto 96-well plates coated with NP-BSA or CGG and detected with biotin-conjugated anti-mouse immunoglobulins. The assays were developed using streptavidin-europium and plates were read on Victor 2 1420 multilabel counter (Perkin Elmer, Waltham, MA, USA). Relative affinities of serum antibodies were evaluated by using altered ligand density ELISA as described earlier [27].

### Flow cytometry analysis

Cell suspensions prepared from spleens excised from mice on day 9 after immunizations were depleted of erythrocytes by ammonium chloride For four-color cell surface staining, 2 × 10^6 ^cells resuspended in PBS containing 2% BSA were incubated with pre-titered dilutions of GL7-FITC, biotinylated anti-IgD, anti-B220-PET-Texas Red, and 2.4 G2-PE for 30 min at 4°C. SA Red 670 was used as a second-step reagent. Cells were analyzed using a FACS Calibur cytometer (Becton-Dickinson, Mountain View, California, USA), and the data were analyzed by FlowJo software (FlowJo, Ashland, Oregon, USA).

### Immunohistochemistry

Spleen isolation, flash freezing, sectioning, and immunohistochemistry were all conducted essentially as described previously [28]. GC numbers were determined by counting the number of PNA^+ ^structures at 10× magnification.

### *In Vivo* Cytotoxicity Assay

C57BL/6 female and male mice, age 8 ~10 weeks, were used for *in vivo *cytotoxicity assays. Female mice were immunized i.p. with syngeneic C57BL/6 male splenocytes (1 × 10^7 ^in 100 μl PBS) at day 0 and boosted at day 10 to generate anti-H-Y cytotoxic T cells. The *in vivo *CTL assay was performed as described earlier with some modifications [29] Target cells were prepared and adoptively transferred to recipient female mice at day 18 post priming and specific killing of target H-Y^+ ^cells was analyzed at day 19. Briefly, male and female C57BL/6 naïve splenocytes were isolated and depleted of erythrocyte with RBC lysis buffer (Sigma-Aldrich, St. Louis, MO, USA). The splenocytes were washed 1× with 10% FBS RPMI 1640 and 2× with PBS. The cell densities were adjusted to 2×10^7 ^cells/ml. Male splenocytes were labeled with a high concentration (5 μM) of 5,6-carboxy-fluorescein succinimdyl ester (CFSE, Molecular Probes, Eugene, OR, USA), and the female splenocyte were labeled with low CFSE (0.5 μM). CFSE labeling was carried out by incubating 2 × 10^7 ^cells/ml in PBS with CFSE at 37°C in a 5% CO2 incubator for 10 min and quenched by adding 10% FBS RPMI to a final volume of 50 ml. Cells were washed 1× with 10%FBS RPMI and 2× with PBS to remove free CFSE. Male and female splenocytes were mixed 1:1 in PBS for the adoptive transfer. 10^6 ^cells in 200 μl PBS were injected into recipient female mice via tail vein injection. Similar amounts of cell were injected into the control unimmunized female mice. Spleens from the recipient mice were harvested after 16 hr, and splenocytes were analyzed for specific killing of H-Y^+ ^target cells by FACS. At least 5,000 CFSE positive cells were analyzed. The recovery and percent killing of the CFSE-labeled targets were calculated as follows:





Data are expressed, as mean ± S.D.

### Measurement of antigen-specific T cell recall responses

Anti-MOG responses were induced in 8 to 12-wk-old mice by immunization with MOG p35–55 in Complete Freund's Adjuvant (CFA). A total of 200 μg of MOG p35–55 peptide and 800 mg of killed *Mycobacterium *was emulsified in CFA and injected S.C. by means of four injections over the flanks. In addition, 200 ng of pertussis toxin dissolved in 200 μl of PBS was injected i.p. at the day of immunization and again the day after [30]. The draining lymph node and spleen were extracted at day 9 and cell proliferation assessed with ^3^[H]-thymidine incorporation upon stimulation with different concentrations of MOG peptide (0, 0.5, 5 and 50 μg/ml). Anti-Ova T cells responses were generated by immunizing the mice with 100 μg of ova in alum in 200 μl volume i.p. The draining lymph nodes and spleen were harvested at day 9 and cells were re-stimulated *in vitro* with varying concentrations of ova (0, 0.1, 0.5 and 1 mg/ml). Proliferation was measured as described above. IL-2 in the supernatant was measured by an R&D Systems ELISA kit according to manufacturer's instructions

### Statistical analysis

Significant differences were determined using one way ANOVA analysis. A p-value of < 0.05 was considered to be statistically significant.

## Results

### Intact T cell-dependent antibody responses in DPP-IV knockout mice

**Figure 1:**
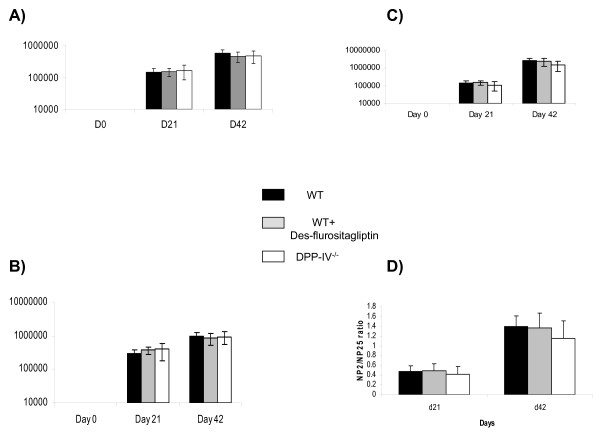
**Primary and secondary immune responses to T cell-dependent antigen are intact in DPP IV null and inhibitor treated mice**. Groups of C57BL/6, DPP IV null and C57BL/6 mice fed with chow containing inhibitor (n = 8) were immunized with NP-CGG in alum and bled various time thereafter and their sera analyzed for anti-NP λ (A), IgG1 (B) or anti-CGG IgG1 (C) titers by ELISA as described in Methods and Materials. The values shown for titers are units defined as EC_50 _of dilution factor × 100. (D) Represents the ratio of anti-NP λ bearing antibody titers obtained on NP_2_-BSA versus NP_25_-BSA coated plates. These data are representative of 3 independent experiments.

The CD26^-/- ^mice were generated as described earlier [23]. Briefly, exons 1 and 2 were replaced with neomycin which led to elimination of CD26 expression on the surface as determined by FACS. There was no DPP-4 enzyme activity detected in the plasma of these mice. DPP-IV knockout animals had no major differences in the leukocytes populations in spleen, thymus and blood as compared to their wild type controls (data not shown). DPP-IV null mice and their wild type littermate control animals were immunized with NP-CGG in alum and the primary humoral immune response was measured at day 21 (Figure 1). The animals were boosted at day 32 with NP-CGG in PBS to measure secondary humoral immune response 10 days later. The primary anti-NP response is dominated by λ bearing antibodies and B cells that make these antibodies are recruited into the memory compartment. These memory B cells secrete high affinity antibodies upon re-challenge with antigen. There were no differences observed in the primary and secondary anti NP λ-responses between the DPP-IV null and wild type control animals (Figure 1A). These antibodies in the secondary responses were predominantly of high affinity as determined by the altered ligand density assay [27] in which antibody titers are assayed by ELISA using different hapten (NP) density capture (Figure 1D). Higher affinity antibodies bind high and low NP-capture plates equally well and hence the ratio of titers is close to 1. Lower affinity antibodies bind better to high NP-coated plates compared to low NP-coated plates and hence the ratio of titers are < 1. There were no differences observed in the dominant anti-NP lambda bearing or IgG1 antibody titers between the two groups of animals during the primary or secondary anti-NP response (Figure 1A &1B). Furthermore affinity matured antibodies produced by memory B cells upon boosting were equivalent between the two groups as measured by the ratio of titers on NP2/NP25. Antibody titers to the carrier protein CGG are dominated by the G1 bearing heavy chain and these were unaltered in the DPP-IV null animals (Figure 1C). All the other isotypes were also unaltered in the DPP-IV null mice as compared to the control animals (data not shown). Equivalent responses were also observed between the CD26 null and control animals immunized with NP-Ova and unconjugated Ova (data not shown). These results indicate intact antigen specific T cell-dependent antibody responses in the DPP-IV^-/- ^animals.

**Figure 2:**
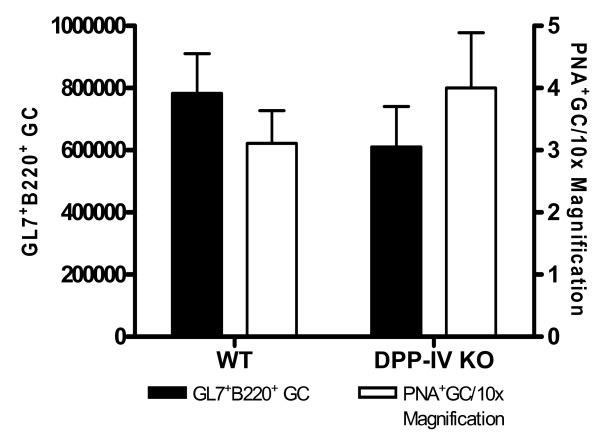
**Germinal center reaction during the T cell-dependent immune response is unaltered in DPP IV knock out animals**. Spleens were extracted on day 9 from animals (n = 3) immunized with NP-CGG in alum. Spleens were weighed and divided into two parts. One part was used for FACS analysis and other for immunohistochemistry. PNA^+ ^GC structures were counted from three random 10× fields/animals. The total numbers of GL-7^+ ^B220^+^IgD^- ^GC cells/spleen were enumerated as described in Materials and Methods. These data are representative of 2 independent experiments.

We also investigated whether germinal center (GC) reactions were dependent on DPP-IV. Germinal centers are secondary lymphoid structures formed in response to antigen and are obligately dependent on the presence of helper T cells and B cells during the anti-NP humoral responses [31]. Splenic GC were evaluated in mice 9 days post immunization. The size of the GC response was evaluated by enumerating GL7^+ ^GC B cells by FACS [32] and the number of germinal centers via IHC (Figure 2). There were no differences in the overall GC reactions as determined by size, frequency (IHC) or numbers of GC B cells (FACS) in DPP-IV^-/- ^mice as compared to control animals. Therefore the data indicate that the T cell-dependent antibody responses in DPP-IV^-/- ^animals are intact.

### Intact T cell-dependent antibody responses in mice treated with a highly selective DPP-IV inhibitor

Pharmacological inhibition of DPP-IV was achieved with a highly selective compound des-fluro sitagliptin (Table 1). Des-fluoro-sitagliptin has properties that are virtually indistinguishable from sitagliptin itself [33]. Indeed, it differs from sitagliptin by only 1 fluorine molecule. This compound does not inhibit DPP-8,-9, Proline dipeptidase, Prolyl endopeptidase, Fibroblast activation protein, Amino peptidase P and Prolidase at >10 μM [15,24] the compound (des-fluro sitagliptin) was milled into rodent chow at ~400 mg/kg. This concentration of compound was sufficient to give >90% inhibition of DPP-IV enzyme activity that was sustained for 24 hours (Table 2). Similar to the DPP-IV^-/- ^animals, the total anti-NP response as measured by λ-bearing and G1 isotype were unaltered in animals with pharmacological inhibition of the DPP-IV enzyme activity (Figure 1A &1B). Moreover, the ability to produce high affinity antibodies from memory B cell stimulation was intact in drug treated animals as observed from the ratios of NP2/NP25 ELISA (Figure 1D). The titers of other isotypes (data not shown) and anti-CGG IgG1 were similar in the drug treated and control animals (Figure 1C). These data therefore show that there are no differences in the amplitude or quality of antibody responses between C57BL/6 controls or drug treated animals. Therefore pharmacological blockade of the DPP-IV enzyme activity has no effect on the primary and secondary T cell-dependent antibody responses to model antigens.

**Table 1:** Properties of Des-flurositagliptin

IC_50 _hDPP-IV	IC_50 _hDPP-8	IC_50 _hDPP-9	IC_50 _mDPP-IV	IC_50 _hDPP-2
27 nM	>10 μM	>10 μM	97 nM	>10 μM

**Table 2:** Des-flurositagliptin dosed at ~400 mpk in feed is able to inhibit the DPP IV enzyme activity for 24 hours in blood.

	**Conversion of substrate in nmol/min**		
	Day 1	Day 2	Day 3
Des-flurositagliptin, am bleed	4.53 ± 0.22	5.44 ± 0.79	6.12 ± 0.48
Des-flurositagliptin, pm bleed	4.90 ± 0.37	8.08 ± 1.8	8.29 ± 1.42
Control, am bleed	39.8 ± 4.0	47.5 ± 5.23	40.1 ± 6.94
Control, pm bleed	31.4 ± 0.65	43.3 ± 6.01	40.3 ± 7.65
DPP-IV^-/- ^mice	1.02 ± 0.51		

The animals were fed chow milled with 6.7 grams of desflurositagliptin in 1 kg of Tekland chow to deliver an effective dose of 400 mpk. Blood were drawn 12 hours apart and DPP IV enzyme activity measured as described earlier. The animals (n = 4 in each group) were maintained on the inhibitor for the entire period of the experiment.

### Intact T cell recall responses in DPP-IV knockout or inhibitor-treated mice

**Figure 3:**
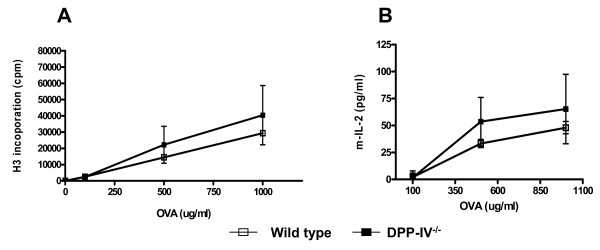
**Ex-vivo T cell recall responses to Ovalbumin are equivalent in DPP IV null and wild type animals**. Wild type (open square) and DPP IV KO (filled square) animals (n = 3) were immunized with Ova in alum and draining mesenteric lymph nodes were harvested on day 9. Cells were than re-stimulated with various concentrations of Ova in vitro and a) proliferation and b) IL-2 production measured by [^3^H] thymidine incorporation and ELISA respectively as described in Methods and Materials. Wild type and DPP-IV KO unstimulated cell proliferation was 843 ± 244 and 908 ± 779 cpm respectively. These data are representative of 2 independent experiments.

Wild type and DPP-IV^-/- ^mice were immunized i.p. with ovalbumin in alum and the draining mesenteric lymph-node T cells were stimulated with various concentrations of Ova *in vitro* (Figure 3). T cells from the DPP-IV^-/- ^mice proliferated in a dose dependent manner to Ova which were similar to that observed in the wild type animals (Figure 3A). In addition, there was no difference observed in the amounts of IL-2 produced between the two groups (Figure 3B).

**Figure 4:**
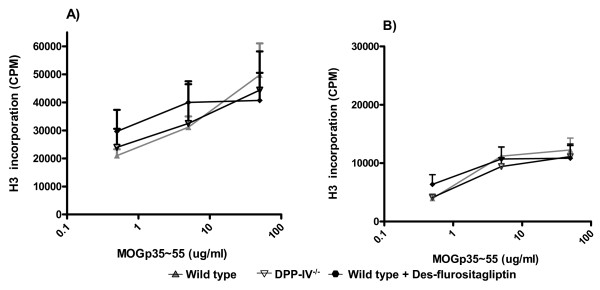
***Ex-vivo *anti-MOG_p35–55 _peptide responses are unaltered in wild type, DPP IV null and des-flurositagliptin treated animals**. Proliferative response to MOG p35–55 of MOG primed T cells from wild-type (filled triangle), DPP IV null (inverted triangle) and des-flurositagliptin (filled diamond) treated animals (n = 4) from draining mesenteric lymph node (A) and spleen (B). Cells were isolated day 11 following immunization with MOG p35–55 in CFA. Cells were cultured with the indicated concentrations of MOG p35–55 for 72 h, and T cell proliferation in each culture was measured using [^3^H] thymidine incorporation assay. The cell proliferation is shown as counts per minute. Proliferation of unstimulated cells from MOG peptide immunized wild type, DPP IV null and wild type+ inhibitor treated mice was 2005 ± 553, 1678 ± 347 and 1876 ± 599 cpm respectively. These data are representative of 2 independent experiments.

Recently, it has been reported that the T cell proliferative recall responses to MOGp35–55 peptide were higher in DPP-IV^-/- ^mice as compared to the wild type controls [30]. In contrast to this report, our data did not reveal any enhancement of T cell responses upon re-stimulation with MOGp35–55 in draining lymph node (Figure 4A) or splenic cells (Figure 4B) in mice with genetic ablation or pharmacological inhibition of DPP-IV activity. This is consistent with our data showing intact T cell recall responses to Ova in DPP-IV^-/- ^mice.

### Intact cytotoxic T cell responses in DPP-IV knockout or inhibitor treated mice

**Figure 5:**
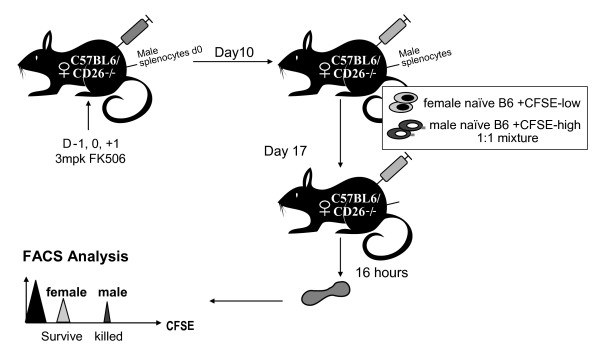
***In vivo *CTL responses protocol in wild type, DPP IV and des-flurositagliptin treated mice**. *In vivo *CD8 T cell responses were generated as outlined in the figure. The female mice were challenged with male cells and female cells differentially labeled with CSFE. Killing of male and female cells was assed by FACS analysis in wild type, DPP IV null and des-flurositagliptin treated animals.

**Figure 6:**
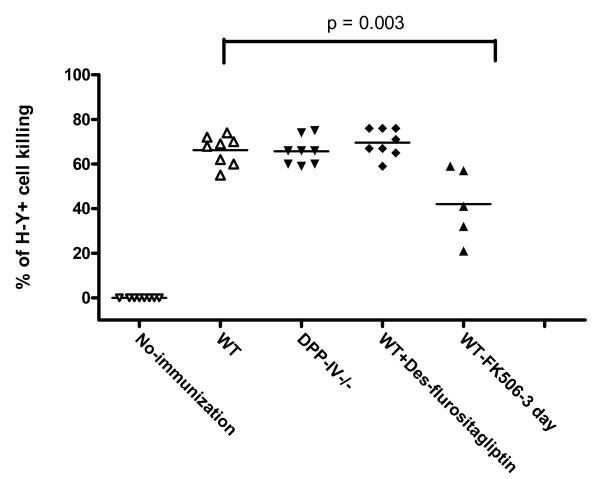
***In vivo *CTL responses are normal in wild type, DPP IV and des-flurositagliptin treated mice**. Specificity of CTL mediated killing was calculated by the formula described in Materials and Methods. FK506 was dosed S.C. for 3 consecutive days starting one day before priming. Des-flurositagliptin was milled in the chow and was given a day before priming and maintained through out the experimental duration (n = 8). These data are representative of 3 independent experiments.

In order to evaluate a potential role for DPP-IV in CD8 T cell function, cytotoxicity was measured using an *in vivo *CTL assay with target cells expressing a minor MHC mismatch (Figure 5). Female mice where primed with the male cells from the same genetic background leading to mismatches in the male (H-Y) antigen expressed on these cells. The primed mice were challenged with a 1:1 mixture of differentially CFSE-labeled male and female cells. The CD8 cytotoxic T cells generated should only kill the male cells and the killing of female cells serves as control to document non-specific cytotoxicity. There were no differences observed in the CD8 T cell cytotoxicity between the wild type, DPP-IV^-/- ^and inhibitor treated wild type animals. All the three groups demonstrated ~67% killing of male cells (Figure 6). As a positive control, FK506, a well characterized T cell immunosuppressant, dosed on (days -1, 0, +1) during priming and boosting showed a statistically significant decrease in CD8 T cell mediated killing of male cells (40%) as compared to untreated wild type animals. These results demonstrate that genetic ablation of DPP-IV or pharmacological inhibition of DPP-IV enzyme activity did not alter CD8 T cell cytotoxicity.

## Discussion

Inhibition of DPP-IV is an effective therapy for type II diabetes. Since this protein is also expressed on the surface of T cells, it is conceivable that DPP-IV inhibitors could have immunomodulatory activity. This possibility was tested by assessing T cell-dependent immune responses in pre-clinical models using both DPP-IV knockout mice and mice treated with a highly selective DPP-IV inhibitor. This inhibitor, des-fluro sitagliptin, has no activity against other dipeptidyl peptidases including DPP-2, DPP-8 and DPP-9 (IC_50_>10 μM). Several distinct types of assays were used to evaluate *in vivo* T cell-dependent responses after antigen challenge including measurements of serum antibody titers, T cell recall responses, and cytotoxic T cell killing activity. We found that loss of DPP-IV activity had no significant effect in any of these T cell-dependent immune response assays.

DPP-IV knockout animals did not show any difference in the kinetics, amplitude or quality of antigen specific antibody responses to a hapten or the protein CGG. In addition, the germinal center reaction, affinity maturation and isotype switching of antibodies, all obligately dependent on T cell help, were similar in wild type and DPP-IV knockout animals. Moreover, treatment of animals with a DPP-IV inhibitor did not alter any of the parameters described above showing that DPP-IV enzyme activity is not essential for mounting T cell- dependent antibody responses.

Recently it has been reported that DPP-IV knockout mice T cell exhibited enhanced proliferative responses to a self peptide MOGp35–55 [30]. Despite using similar experimental procedures, our results failed to reproduce these observations in two independent experiments. In addition, treatment of mice with the DPP-IV inhibitor, des-fluro sitagliptin, did not have any significant effect on MOG-specific T cell recall responses. The reason for this discrepancy is uncertain. Of note, the lack of effect of DPP-IV inhibition on MOG-specific immune responses is similar to results seen for other protein antigens such as CGG and Ova. These results strongly suggest that DPP-IV does not play a significant role in T cell recall responses to protein antigens.

CD8 T cells play an important role in immunity against intracellular pathogens. We investigated the effects of DPP-IV blockade on *in vivo* cytotoxic T cell functions by challenging the female mice with male cells to generate H-Y specific cytotoxic T cells. The primed animals challenged with male cells demonstrated equivalent cytotoxicity when the DPP-IV expression was genetically ablated or the DPP-IV enzyme activity blocked with des-fluro sitagliptin in comparison to the wild type animals (Figure 6). These results indicate that the CD8 T cell cytotoxic function is intact in animals with DPP-IV blockade.

Our data add to and extend the earlier observation that the DPP-IV enzyme activity is not required for T cell activation or co-stimulating properties *in vitro  *[8,34-37]. Busso *et al *have observed no effects of CD26 deficiency on *in vitro* or *in vivo* proliferation of lymph node and spleen cells nor on *in vivo *antibody response to mBSA immunization [38]. Furthermore, although lower IgG1 and IgG2a titers were found in CD26 knockout animals as compared to wild type mice immunized with poke weed mitogen (PWM) in the primary response [10] these differences were eliminated upon boosting with PWM. Earlier reports assessing the effect of DPP-IV on T cell functions have used inhibitors that were non-selective against other dipeptidyl peptidases [16-18,21,39]. The compound des-fluro sitagliptin used in the current studies is structurally similar to sitagliptin, a highly selective DPP-IV inhibitor that is approved for clinical use in type II diabetic patients. We have previously shown that highly selective DPP-IV inhibitors do not impair T cell activation *in vitro*, in contrast to less selective inhibitors that cross-inhibit DPP8 and DPP9 [15]. The current studies extend these observations by showing that highly selective inhibition of DPP-IV does not disrupt T cell responses *in vivo*.

While DPP-IV does not appear to affect T cell-dependent responses, this protein could modulate other types of immune responses. For example, in humans, DPP-IV is reported to associate with adenine deaminase (ADA) on the surface of the T cell and could prevent the inhibitory effects of adenosine on T cell proliferation [40]. The crystal structures of both the sitagliptin/DPP-IV complex and the ADA/DPP-IV complex have been solved. While sitagliptin binds DPP-IV to inhibit its enzymatic activity [41], this binding does not alter the overall conformation of the DPP-IV molecule, including the aspect of the molecule that binds to ADA [42]. Therefore, sitagliptin is highly unlikely to alter the ADA-binding properties of DPP-IV. However, since the CD26 molecule does not associate with ADA in mice its impact on T-cell function cannot be addressed in rodents [43]. DPP-IV has also been reported to associate with CD45 and could thereby modulate signal transduction [44]. We did not directly address functions that could be altered by disrupting the cell surface association of CD45 with DPP-IV. Of note, inhibition of DPP-IV enzymatic activity would not be expected to affect the association of DPP-IV with other cell surface proteins. Our studies focus on the role of DPP-IV in *in vivo* T-cell functions. Several studies including work done by Morimoto *et al* has implicated potentially diverse roles for DPP-IV in human T-cell functions. For instance expression of CD26 on T-cells has been associated with co-stimulatory functions [45]. Studies performed by this group have used isolated T-cells stimulated *in vitro* in a variety of ways (including with anti-CD26 antibody, recombinant soluble CD26, and an Fc-caveolin-1 fusion protein). Indeed, the more recent results from this group focus on dissecting *in vitro* protein-protein interactions and signaling by the CD26 molecule and its putative ligand, caveolin-1. DPP-IV was reported to have co-stimulatory activity in studies using isolated T-cells or T-cell lines stimulated by cross-linking surface CD26 with antibodies [46]. However, the physiological relevance of these studies is uncertain, e.g., endogenous cross-linking ligands have not been identified and, clearly, there is much work to be done to further establish the potential physiologic role that CD26 might play in immune processes.

Isolated human Th1 cells expressed three to six fold more CD26 protein than Th2 cells and were more responsive to anti-DPP-IV cross-linking [47]. Interestingly, DPP-IV enzyme activities were equivalent in both Th1 and Th2 cells. DPP-IV could have different role in distinct T-cell populations. The potential role of DPP-IV in other T-Cell subsets Th17, Treg requires further studies.

It has been previously shown that DPP-IV is capable of *in vitro* cleavage of SDF-1α and a number of other chemokines like CXCL6, CXCL9, CXCL10, CXCL11, CCL3L1, CCL5, CCL11 and CCL22 *in vitro  *[7,38,48-54]. Recently it was shown that DPP8 could also cleave CXCL10, CXCL11 and CXCL12 *in vitro *[55]. However, whether cleavage of these chemokines occurs *in vivo* has yet to be demonstrated and thus the physiological relevance is uncertain. DPP-IV^-/- ^mice were found to be resistant to G-CSF mediated mobilization of hematopoietic stem cell, suggesting a potential role for DPP-IV in regulating SDF-1α in the bone marrow [38,48]. This has led to the postulation that DPP-IV inhibitors could be clinical useful for bone marrow transplantation.

## Conclusion

The manuscript provides important data on the role of DPP-IV on T-cell immune function. Genetic ablation of DPP-IV or pharmacological inhibition of its enzymatic activity had no measurable effect on T cell-dependent immune responses in mice. While there may be limitations regarding extrapolation of the results to humans, these results are consistent with the favorable clinical experience with sitagliptin, to date.

## Authors' contributions

KV was involved in conception, design, and analysis, interpretation of experiments, drafting and revision of the manuscript. GP was involved in designing, data acquisition and interpretation of humoral immune response experiment, RP for design, data acquisition and interpretation of T-cell recall and *in vivo* T-cytotoxic assays. YC contributed to design, data acquisition and interpretation for germinal center and immunoassays, KP ran the DPP IV enzyme assays, GE provided pharmacological support for the compound studies. DZ had input in conception, design and revising it critically for intellectual content. All authors read and approved the final manuscript.
